# Phylogenetic annotation of *Caenorhabditis elegans* heat shock protein 70 genes

**DOI:** 10.17912/micropub.biology.000633

**Published:** 2022-09-01

**Authors:** Pirzada Hasnain, Gen Kaneko

**Affiliations:** 1 University of Houston-Victoria, Victoria, TX, United States.

## Abstract

Annotation of the 70 kDa heat shock proteins (Hsp70s) has been chaotic especially in invertebrates. In this study, we validated an emerging nomenclature of Hsp70s, which can be potentially applied to all metazoan Hsp70s, by conducting a genome-wide annotation of *Caenorhabditis elegans* Hsp70s. Using the phylogenetic annotation, the seven canonical *C. elegans* Hsp70s were successfully classified into four known lineages, cytosolic A, cytosolic B, endoplasmic reticulum, and mitochondria. Motifs specific to each lineage were all conserved in the *C. elegans* Hsp70s. From these results, we propose new aliases of *C. elegans* Hsp70s that should help future annotation of this important molecular chaperone.

**Figure 1. Phylogenetic annotation of Caenorhabditis elegans 70 kDa heat shock protein (Hsp70) genes:**
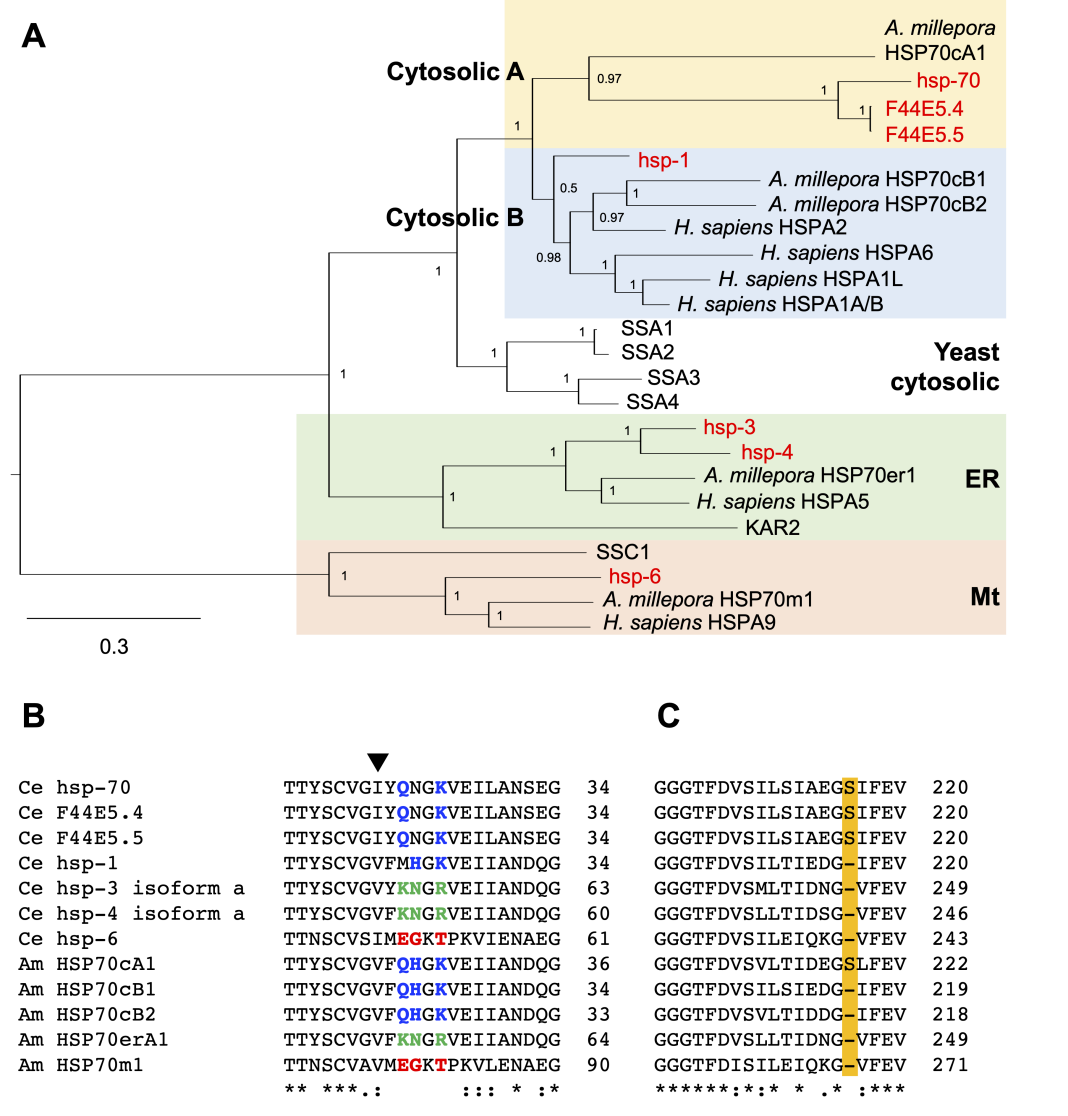
(A) Bayesian phylogenetic tree constructed using the LG + I + G model (M-Coffee alignment, best model according to BIC). Deduced amino acid sequences were used, and the mitochondrial lineage was set as the root. Numbers indicate the posterior probability support for each node. *Saccharomyces cerevisiae* SSA1 (NP_009396.2), SSA2 (NP_013076.1), SSA3 (NP_009478.1), SSA4 (NP_011029.3), KAR2 (NP_012500.3), and SSC1 (NP_012579.1); Coral *Acropora millepora* HSP70cA1 (XP_029210797.1), HSP70cB1 (XP_029206535.1), HSP70cB2 (XP_029194855.1), HSP70erA1 (XP_029187601.1), HSP70mA1 (XP_029208733.1); *Homo sapiens* HSPA1A/B (NP_005337.2), HSPA1L (NP_005518.3), HSPA2 (NP_068814.2), HSPA5 (NP_005338.1), HSPA6 (NP_002146.2), and HSPA9 (NP_004125.3). (B, C) M-coffee alignment of *C. elegans* (Ce) and *A. millepora* (Am) Hsp70s. Residues characteristic to the cytosolic, endoplasmic reticulum, and mitochondrial lineages are shown in blue, green, and red, respectively. The HSP70cA-specific serine residues are highlighted. The potential target of gene conversion is indicated by a black triangle. Numbers on the right of each panel indicate amino acid numbers from the N-terminus.

## Description

The 70 kDa heat shock proteins (Hsp70s) play important roles in various biological processes. Traditionally, the Hsp70 family members have been classified into stress-inducible Hsp70s and constitutive heat shock cognates (Hsc70s). This nomenclature has been revised in human Hsp70s more than a decade ago (Kampinga et al. 2009), and human Hsp70s are currently called HSPA1, HSPA2, etc. without the expression information in their names. This revision is reasonable because there is no clear distinction between “stress-inducible” and “constitutive” expressions. For example, human HSPA8 (Hsc70) used to be described as a constitutive gene (Dwornczak and Mirault 1987), but it is now widely accepted that this gene is upregulated by cellular stresses including inflammation and infection (Stricher et al. 2013). Nothing is surprising about these discrepancies because Hsp70/Hsc70 promoters contain multiple regulatory elements (Garbuz 2017).

The human Hsp70 nomenclature has been sometimes used to annotate newly-identified Hsp70s in other organisms, but this is often inappropriate according to the molecular evolution of metazoan Hsp70s. Namely, canonical metazoan Hsp70s are classified into four lineages, cytosolic A, cytosolic B, endoplasmic reticulum (ER), and mitochondria (Mt) (Yu et al. 2021). The gene duplications that gave rise to the four lineages are ancient, but many human cytosolic Hsp70s (HSPA1A/B, HSPA1L, HSPA2, HSPA6, and HSPA8) resulted from recent duplications specific to the phylum Chordata and/or subphylum Craniata (Wada et al. 2006; Yu et al. 2021). Therefore, many invertebrate cytosolic Hsp70s should be equally orthologous to all human cytosolic Hsp70s and cannot be named using just one of them. i.e., a name like “organism + HSPA2” is problematic if the organism is phylogenetically distant from human — the name should be like “organism + HSPA1A/1B/1L/2/6/8” because the duplications took place after divergence of human and the organism of interest. Unfortunately, the questionable annotation “organism + a single human HSPA name” is quite prevalent in invertebrate studies (Cheng et al. 2016; McKinstry et al. 2017), while some studies appropriately grouped all human cytosolic Hsp70s to annotate invertebrate Hsp70s (Hu et al. 2019; Shiel et al. 2015). There is an urgent need for developing an adequate nomenclature that correctly represents the nature of metazoan Hsp70s, which is based on neither expression patterns nor the human nomenclature.

We recently proposed a new nomenclature for metazoan Hsp70s based on the above-mentioned four lineages (Yu et al. 2021): “Hsp70 + subcellular localization + linage + copy number in the organism” e.g., Hsp70cA1 for cytosolic Lineage A, copy 1. In this study, we described the copy number as “copy 1” instead of adding the digit at the end to clarify the relationship between orthologues. This annotation strategy was successfully applied to the genome-wide screening of Hsp70s in the rotifer *Brachionus plicatilis* sensu stricto (Grewal et al. 2022), an annelid *Urechis unicinctus* (Liu et al. 2022), and *D. melanogaster* (Kaneko 2022) as well as has been positively cited to describe Hsp70s in various fields (Aksakal and Ekinci 2021; Khieokhajonkhet et al. 2022; Schnebert et al. 2022; Stamperna et al. 2021). The latest update of the FlyBase (FB2022_03 released on June 9, 2022) incorporated the proposed *D. melanogaster* Hsp70 gene names. The aim of the present study is to further validate the robustness of the novel annotation strategy using Hsp70s of the nematode *Caenorhabditis elegans* .

The gene class hsp of *C. elegans* (WormBase version WS284, ID: hsp) contained 24 members, including 9 *hsp70* -like genes ( *hsp-1* to *hsp-6* , *hsp-70* , *hsp-75* , and *stc-1* ) (Table 1). Other members of this gene class included *hsp-110* , *hsp-90* , *hsp-60* , and many small Hsp genes. We also searched the WormBase for *C. elegans* Hsp70 genes using “hsp70” as a keyword. This search resulted in 25 hits, and manual inspection of the result further identified four uncharacterized *hsp70* -like genes, F44E5.4, F44E5.5, T14G8.3, and T24H7.2 (Table 1). The F44E5.4 and F44E5.5 genes are clustered on the *C. elegans* genome and share 100% amino acid identity, suggesting a recent gene duplication event. Deduced amino acid sequences of the 13 *hsp70* -like genes (Table 1) were subjected to the preliminary phylogenetic analysis.

The preliminary phylogenetic tree showed that T14G8.3, T24H7.2, *stc-1,* and *hsp-75* do not encode canonical HSP70s. i.e., these proteins diverged earlier than the split of the four lineages. The subsequent web BLAST search indicated that T14G8.3 and T24H7.2 encode the hypoxia up-regulated 1 (HYOU1) protein, and *stc-1* encodes HSP70-13 (a distant member of the HSP70 family, the function of which largely remains unknown). The BLAST top hits of *hsp-75* were mostly uncharacterized proteins. These genes were therefore excluded from the following analysis.

Table 1. *Caenorhabditis elegans* Hsp70 genes in NCBI and WormBase version WS284

**Table:** 

Gene	Sequence	WormBase ID	Proposed alias	Remarks; identity to NCBI genes at the amino acid level
*hsp-1*	F26D10.3	WBGene00002005	hsp70cB	100% to NP_503068.1
*hsp-2*	N.A.	WBGene00002006	–	Pseudogene
*hsp-3*	C15H9.6a	WBGene00002007	hsp70er (copy 1)*	Two isoforms, a and b. Isoform a is 100% identical to NP_001370435.1, 97.6% to AAA28074.1.
*hsp-4*	F43E2.8a	WBGene00002008	hsp70er (copy 2)*	Two isoforms, a and b. Isoform a is 100% identical to NP_001370395.1.
*hsp-5*	N.A.	WBGene00002008	–	Probable pseudogene
*hsp-6*	C37H5.8	WBGene00002010	hsp70m	100% to NP_504291.1
*hsp-70*	C12C8.1	WBGene00002026	hsp70cA (copy 1)*	100% to NP_492485.1
*hsp-75*	R151.7	WBGene00020110	–	100% to NP_741220.2
*stc-1*	F54C9.2	WBGene00006059	–	100% to NP_495808.2. Likely encodes HSP70-13, predicted molecular weight 49 kDa.
F44E5.4	F44E5.4	WBGene00009691	hsp70cA (copy 2)*	100% to NP_496509.1.
F44E5.5	F44E5.5	WBGene00009692	hsp70cA (copy 3)*	100% to NP_496509.1.
T14G8.3	T14G8.3	WBGene00011771	–	Likely encodes HYOU1, predicted molecular weight 105 kDa.
T24H7.2	T24H7.2	WBGene00020781	–	Likely encodes HYOU1, predicted molecular weight 104 kDa.

N.A., not available; *Genes with multiple copies are registered with copy numbers in WormBase (i.e., hsp70er1, hsp70er2, hsp70cA1, hsp70cA2, and hsp70cA3).

To annotate the remaining seven *C. elegans hsp70* -like genes, we constructed six phylogenetic trees using three alignment methods (Clustal Omega, MUSCLE, and M-Coffee) and two tree-building methods (Bayesian and maximum-likelihood). The topology of these phylogenetic trees was very similar as also demonstrated in our previous studies (Grewal et al. 2022; Kaneko 2022; Yu et al. 2021). In a representative phylogenetic tree (Fig. 1A), *C. elegans* Hsp70s were separated into cytosolic A, cytosolic B, ER, and Mt lineages. In each lineage, yeast Hsp70s were basal to other metazoan Hsp70s, confirming that the separation of these lineages was ancient. *C. elegans* Hsp70s diverged before the split of coral and human Hsp70s, which is consistent with the suggested phylum-specific gene evolution (Yu et al. 2021). In the phylogenetic tree, *C. elegans* hsp-70, F44E5.4, and F44E5.5 belonged to the cytosolic A lineage, hsp-1 belonged to the cytosolic B lineage, hsp-3 and -4 belonged to the ER lineage, and hsp-6 belonged to the Mt lineage.

These *C. elegans* Hsp70s contained Hsp70 signature motifs (Yu et al. 2021) that supported the phylogenetic annotation. First, the lineage-specific motifs were well conserved in all Hsp70s (Fig. 1B). Second, the cytosolic A lineage-specific extra serine residue was present only in hsp-70, F44E5.4, and F44E5.5, which were classified into this lineage in the phylogenetic analysis (Fig. 1C). Third, the hsp-3 and hsp-4 had the ER-retention signal KDEL and HDEL, respectively, at the C-terminal. These results indicate that canonical *C. elegans* Hsp70s have followed the same evolutionary paths as other metazoan Hsp70s, allowing us to classify them using the emerging phylogenetic annotation method. In contrast, the valine and isoleucine residue (indicated by a black arrowhead in Fig. 1B) had a variation that was not consistent with the lineages. *C. elegans* hsp-1 (Cyt B), hsp-3 (ER), and hsp-4 (ER) had isoleucine, whereas other Hsp70s had valine, at this position. It is possible that this region contains homoplasy or has undergone gene conversions after the ancient duplications. Such an event may explain the low support for hsp-1 belonging to the Cyt B lineage.

A limitation of the phylogenetic annotation is that current phylogenetic methods cannot quantitatively evaluate the effect of gene conversion, a type of homologous recombination between paralogues in a genome. Hsp70 sequences have generally been homogenized by gene conversion at least in *D. melanogaster* (Bettencourt and Feder 2002), nematode (Nikolaidis and Nei 2004), and human (Kudla et al. 2004), which may have affected the topology of the phylogenetic tree. The effect must be minor as long as the gene conversion has not altered the entire gene, thus allowing the phylogenetic programs to identify the gene origin. However, if a gene conversion that covers the entire gene has completely deleted the previous gene, the origin of the previous gene cannot be determined by the phylogenetic analysis. It is also noted that the distance in our phylogenetic tree may not accurately represent the divergence time between paralogues such as *C. elegans* hsp-3 and hsp-4 because of gene conversion.

In conclusion, the present study successfully validated the robustness of the phylogenetic annotation using *C. elegans* Hsp70 family members, identifying two poorly characterized Hsp70-like proteins (F44E5.4 and F44E5.5) as Hsp70cA. Currently there is no strong need to replace the official *C. elegans* Hsp70 names with the newly proposed names in Table 1, but it should be worth to include the proposed names and gene origins in the “Other names” and “Gene description” sections in the WormBase, respectively, as already have done in the FlyBase for *D. melanogaster* Hsp70s. These new names will help researchers to pay attention to the molecular evolution of the Hsp70 family members, allowing us to avoid the use of expression patterns and the human nomenclature in the gene/protein names of other organisms in future Hsp70 studies.

## Methods

Sequences were obtained from the WormBase version WS284. Sequences were analyzed as reported previously (Kaneko 2022). Briefly, Clustal Omega (clustalo v1.2.4) (Sievers et al. 2011), M-Coffee (Wallace et al. 2006), and MUSCLE v3.8.1551 were used for sequence alignment. Best-fit models were determined for each tree by ProtTest-3.4.2 (Darriba et al. 2011) using the Bayesian information criterion. Bayesian and maximum-likelihood trees were constructed using MrBayes 3.2.7a (Ronquist et al. 2012) and MEGA11, respectively, using the same setting as our previous study (Kaneko 2022). FigTree (v1.4.4) was used to visualize the phylogenetic trees.
